# The dose-response relationship for cardiovascular disease is not necessarily linear

**DOI:** 10.1186/s13014-017-0811-2

**Published:** 2017-04-27

**Authors:** Uwe Schneider, Marina Ernst, Matthias Hartmann

**Affiliations:** 1 Department of Physics, Science Faculty, University of Zürich, Zürich, Switzerland; 2 Radiotherapy Hirslanden, Witellikerstrasse 40, CH-8032 Zürich, Switzerland

**Keywords:** Dose-response curves, Cardiovascular disease, Bio-physical modeling, NTCP

## Abstract

The probability for a complication after radiotherapy is usually a function of dose and volume in the organ or tissue of interest. In most epidemiological studies the risk for a complication is stratified in terms of dose, but not irradiated volume. We show that the obtained risk cannot generally be applied to radiotherapy patients.The epidemiological data of Darby et al. (N Engl J Med 368:2527, 2013) who found a linear relationship between the excess relative risk of major coronary events as function of mean heart dose in patients treated with tangential breast irradiation are analyzed. We have used the relative seriality model for a partly irradiated heart (“a lot to a little”) which models radiation therapy using two tangential fields. The relative seriality model was then used to predict NTCP of cardiovascular disease for a homogenously irradiated heart (“a little to a lot”). The relative seriality model was fitted to the data of Darby et al. (N Engl J Med 368:2527, 2013) for tangential breast irradiation. For the situation “a little to a lot” it was found that the dose-response relationship is sigmoidal and contradicts the findings of Darby et al. (N Engl J Med 368:2527, 2013). It was shown in this work that epidemiological studies which predict a linear dose-response relationship for cardiovascular disease can be reproduced by bio-physical models for normal tissue complication. For irradiation situations which were not included in the epidemiological studies, e.g. a homogenous irradiation of the heart (“a little to a lot”) the dose-response curve can be different. This could have consequences whether or not IMRT should be used for treating breast cancer. We believe that the results of epidemiological studies should not be generally used to predict normal tissue complications. It is better to use such data to optimize bio-physical models which can then be applied (with caution) to general treatment situations.

## Background

The results from epidemiological studies of side effects from radiation therapy can provide valuable information for the decision-making process in clinical treatment planning and can ultimately help to treat a patient successfully with radiation while minimizing adverse reactions. However, epidemiologically obtained data describe a side effect for a well-defined treatment situation and cannot be generally applied to all radiotherapy patients. The application of specifically obtained dose-response relationships to other treatment techniques/modalities requires the extrapolation of the obtained risks. This extrapolation can be achieved by the use of biophysical models.

The probability for a complication is usually a function of dose and volume in the organ or tissue of interest. In most epidemiological studies the risk for a complication is stratified in terms of dose, but not irradiated volume. We are concerned that this data, investigated for a very specific dose-volume situation, is used in clinical practice in a generalized way for completely different clinical settings.

In this report we want to point out the pits and pearls by using as an example the rate of major coronary events after radiation therapy. In particular we analyze the epidemiological data of Darby et al. [1] who found a linear relationship between the excess relative risk (ERR) of major coronary events as function of mean heart dose in patients treated with tangential breast irradiation. They obtained a risk factor of 7.4% per Gy of mean dose to the heart which is currently widely used in clinical practice. We believe that the general usage of this factor is dangerous, since it is a greatly simplified representation of complex biological processes which can only be applied safely to exactly the same situation for which the data were obtained (tangential breast irradiation). For this purpose we show that if the epidemiological data are used as input into a bio-physical model describing cardiovascular complications, the shape of the resulting dose-response relationship is highly dependent on the specific dose-volume situation. We believe further that the data of Darby et al. [1] should be better used for verifying and optimizing existing NTCP models of coronary events which can then be used in a more general setting.

## Main text

Darby et al. [1] conducted a population-based case–control study of major coronary events (i.e., myocardial infarction, coronary revascularization, or death from ischemic heart disease) in 2168 women who underwent radiotherapy for breast cancer between 1958 and 2001 in Sweden and Denmark. The study included 963 women with major coronary events and 1205 controls. For each woman, the mean radiation doses to the whole heart were estimated from her radiotherapy chart. They found that excess rates of major coronary events increased linearly with the mean dose to the heart by 7.4% per Gy. This obtained dose response-relationship for major coronary events is illustrated by the symbols in Fig. 2 with the corresponding 95% confidence interval as a function of mean heart dose. The results of Darby et al. were validate by van den Bogaard et al. [2] in an independent cohort using individual 3D CT planning data.

### Probability of a complication is function of dose and volume

**Fig. 1:**
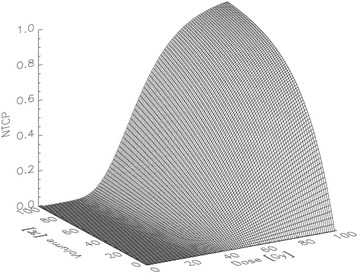
Normal tissue complication probability (NTCP) for cardiac mortality from ischemic heart disease and myocardial infarction modeled with the relative seriality model [4] resulting in the model parameters D50 = 52.3 Gy, γ = 1.28 and s = 1 as obtained by Gagliardi et al. [3]

The probability of a complication in radiation therapy is usually a function of dose and irradiated volume. The complex relationship between heart complication, dose and volume is illustrated in Fig. 1. Here the normal tissue complication probability (NTCP) for cardiac mortality from ischemic heart disease and myocardial infarction was taken from Gagliardi et al. [3] and modeled with the relative seriality model [4] resulting in the model parameters D50 = 52.3 Gy, γ = 1.28 and s = 1. The tangential breast treatments, which were analyzed by Darby et al. [1], were dosimetrically reproduced by assuming a simplified geometry of the heart. If it is assumed that the heart as a spherical volume which is irradiated partly with the treated dose *D*_*T*_ = 60 Gy, then the % of irradiated heart volume *V*_*H*_ is related to mean heart dose: *V*_*H*_ *= D*_*mean*_ */D*_*T*_. The relative seriality model yields then NTCP as a function of mean heart dose:

1 $$ \boldsymbol{NTCP}={\left(\mathbf{1}-{\left(\mathbf{1}-\boldsymbol{P}{\left({\boldsymbol{D}}_{\boldsymbol{T}}\right)}^{\boldsymbol{s}}\right)}^{{\boldsymbol{V}}_{\boldsymbol{H}}}\right)}^{\mathsf{1}/\mathsf{s}}/{\boldsymbol{R}}_{\boldsymbol{base}}, $$

with

2 $$ P\left({D}_T\right) = {2}^{-\boldsymbol{exp}\left(\boldsymbol{e}\cdot \boldsymbol{\gamma} \cdot \left(1-\frac{D_T}{D50}\right)\right)}. $$

Usually NTCP is given in absolute quantities. However, to be able to compare NTCP with the relative results of Darby et al. [1], we divide in Eq. 1 the original NTCP by *R*_*base*_= 0.38% , which is the base line risk for major coronary events and was taken from Table S11 of [1] for the age at exposure ﻿(59.6 years) and attained age (67.1 years) of the cohort analyzed by Darby et al. [1].

The ERR from Darby et al. [1] were then fitted to Eqs. 1 and 2. The data fits were produced with a least-squares algorithm with the software package PVWave (PV-Wave Advantage, PV-Wave Command Language, Version 9.01 - Numerics, Inc - 2008) using the epidemiological errors as weights. The model parameters *D*_*50*_, *γ* and *s* were obtained.

### Fit of the Darby data to “a lot to a little”

**Fig. 2:**
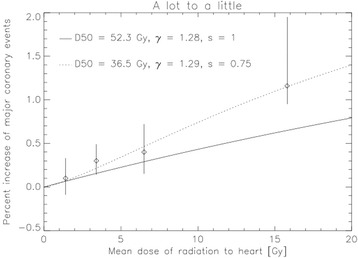
The dose-response curve of the situation “a lot to a little” using the relative seriality model based on the model parameters of Gagliardi et al. [3] shown as the solid line (D50 = 52.3 Gy, γ = 1.28 and s = 1.0). The fit to Darby’s data (symbols) is shown as the dotted line with the model parameters D50 = 36.5 Gy, γ = 1.29 and s = 0.7

The dose-response curve from the relative seriality model based on the model parameters of Gagliardi et al. [3] is shown as the solid line in Fig. 2 (*D*_*50*_ = 52.3 Gy, *γ* = 1.28 and *s* = 1.0). This dose-response curve represents the situation “a lot to a little”, where the parts of the heart in the tangential treatment beams receive the treatment dose and the rest of the heart more or less no dose. The NTCP model shows qualitatively a linear dependence on mean heart dose as it was observed by Darby et al. [1]. Quantitatively the model can be fitted to the Darby data which results in the dotted line with the model parameters *D*_*50*_ = 36.5 Gy, *γ* = 1.29 and *s* = 0.75.

### Model results for “a little to a lot”

**Fig. 3:**
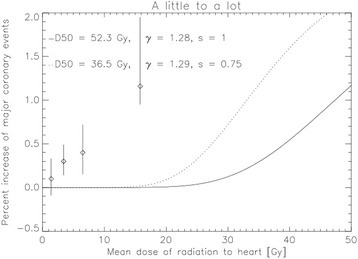
The dose-response curve of the situation “a little to a lot” from the relative seriality model based on the model parameters of Gagliardi et al. [3] as the solid line (D50 = 52.3 Gy, γ = 1.28 and s = 1.0).). The fit to Darby’s data (symbols) is shown as the dotted line with the model parameters D50 = 36.5 Gy, γ = 1.29 and s = 0.75

We used the NTCP model based on the Gagliardi parameters (solid line) and the parameters obtained from the Darby data (dotted line) to calculate NTCP for a homogenous dose distribution as shown in Fig. 3. It is obvious that risk as a function of mean dose for a homogenously irradiated heart is not linear. In fact the dose-response curve exhibits the typical sigmoidal behavior with a negligible risk for major coronary events up to a dose of 15 Gy. For this reason the risk of 7.4% per Gy determined by Darby et al. [1] which is also shown in Fig. 3 as the symbols cannot be used in situations where the heart is irradiated “a little to a lot” and would strongly overestimate the risk of major cardiac events.

### Discussion

**Fig. 4:**
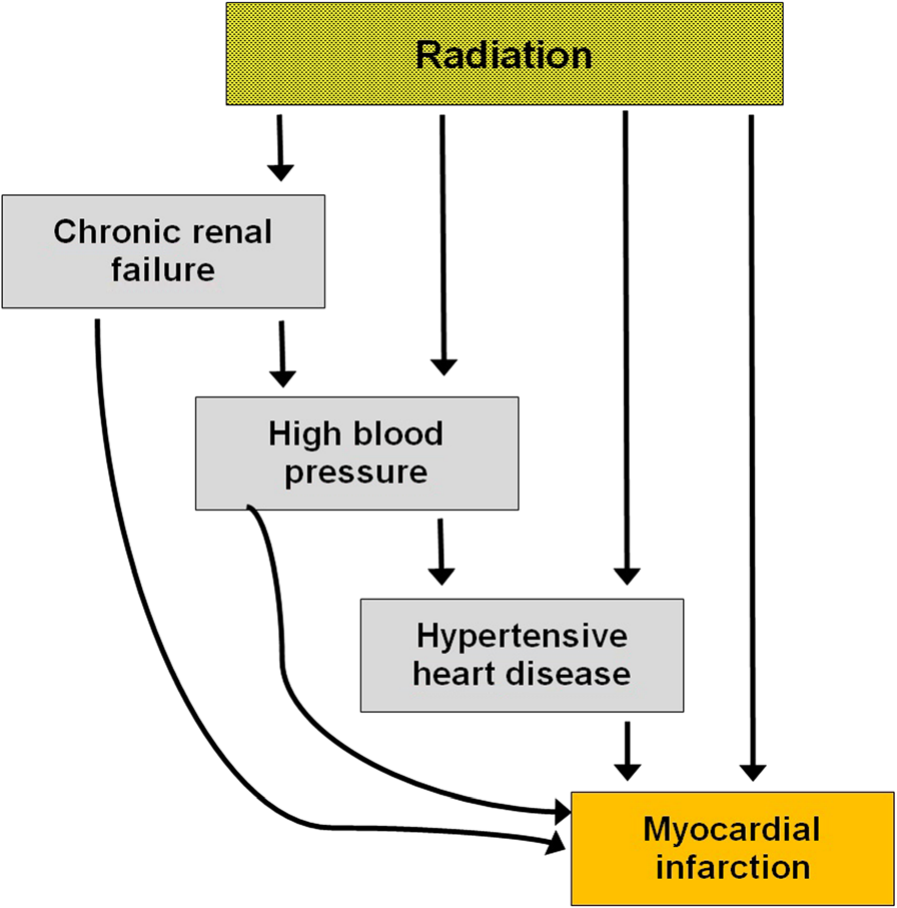
Illustration of different pathways from radiation to heart disease in the atomic bomb survivors cohort as proposed by Adams et al. [6]

It must be noted that the epidemiological life span study of the A-bomb survivors shows a significant quadratic dose-response relationships for hypertension and for myocardial infarction among survivors exposed at less than 40 years of age [5]. As the A-bomb survivors were irradiated with a more or less homogenous doses smaller than 3 Gy it corresponds to the scenario (“a little to a lot”). At a first glance these findings contradict our results illustrated in Fig. 3. However, the A-bomb survivors received a whole body radiation exposure and, since radiation dose is also associated with increased hypertension incidence, it can be suggested that radiation dose may be associated with chronic renal failure, thus explaining part of the mechanism for increased cardiovascular disease. Indeed Adams et al. [6] have shown that part of the risk of cardiovascular disease, particularly myocardial infarction risk, is mediated by renal dysfunction which is a result of the whole body irradiation (see Fig. 4). This relationship between renal dysfunction and cardiovascular disease is usually not present in radiotherapy, which is typically a local treatment.

It should also be noted that Ghobadi et al. [7] have shown that irradiation of heart, lung, or both independently induces specific cardiac dysfunction. These results show that treatment of the lung with radiation therapy can enhance cardiac toxicity through an unknown mechanism which is not yet included in bio-physical models.

## Conclusions

It was shown in this work that epidemiological studies which predict a linear dose-response relationship for cardiovascular disease can be reproduced by bio-physical models for normal tissue complication. We have used the relative seriality model for a partly irradiated heart (“a lot to a little”) which models radiation therapy using two tangential fields. It was also shown that the model predicts a sigmoidal dose-response curve - and not a linear one - for cardiovascular disease in the situation of a homogenously irradiated heart (“a little to a lot”). This has direct consequences for the application of new treatment techniques, e.g. the use of the IMRT technique for treating the breast. IMRT is reducing the volume irradiated at high doses and may therefore result in more favorable cardiac outcomes than treating with tangents if the heart cannot be removed from the tangent by, e.g., deep inspiration breath hold techniques (DIBH). This is supported by a recent publication of Zagar et al. [8] who found in a prospective study that tangential breast treatment with DIBH for left-sided breast cancer is an effective means to avoid early RT-associated cardiac perfusion defects.

We believe that the results of epidemiological studies should not generally be used to predict normal tissue complications. It is better to use such data to optimize bio-physical models which can then be applied (with caution) to general treatment situations.
